# The association of urinary sodium excretion and the need for renal replacement therapy in advanced chronic kidney disease: a cohort study

**DOI:** 10.1186/s12882-016-0338-z

**Published:** 2016-09-05

**Authors:** Andrea Mazarova, Amber O. Molnar, Ayub Akbari, Manish M. Sood, Swapnil Hiremath, Kevin D. Burns, Timothy O. Ramsay, Ranjeeta Mallick, Gregory A. Knoll, Marcel Ruzicka

**Affiliations:** 1 Division of Nephrology, Department of Medicine, The Ottawa Hospital, Ottawa, Canada; 2 Division of Nephrology, Department of Medicine, McMaster University, Hamilton, Canada; 3 Kidney Research Centre, Ottawa Hospital Research Institute, Ottawa, Canada; 4 Clinical Epidemiology Program, Ottawa Hospital Research Institute, Ottawa, Canada

**Keywords:** Chronic kidney disease, Sodium intake, Urinary sodium excretion, eGFR decline

## Abstract

**Background:**

Restriction of dietary sodium is routinely recommended for patients with chronic kidney disease (CKD). Whether or not sodium intake is associated with the progression of CKD and mortality remains controversial. We evaluated the association of urinary sodium excretion (as a surrogate for sodium intake) on the need for renal replacement therapy and mortality in patients with advanced CKD.

**Methods:**

We conducted a retrospective study of patients followed at a CKD clinic of a tertiary care hospital from January 2010 to December 2012. Adult patients with advanced CKD (estimated glomerular filtration rate (eGFR) <30 ml/min/1.73 m^2^) were included. Using a time-to-event analysis, we examined the association of urinary sodium excretion as a continuous and also as a categorical variable (categorized as low sodium diet - LSD (<100 mEq/day), medium sodium diet - MSD (100–150 mEq/day), and high sodium diet - HSD (>150 mEq/day) and the outcomes of interest. The primary outcome was defined as composite of progression to end-stage renal disease requiring any type of renal replacement therapy and mortality. The secondary outcome was change in eGFR/year.

**Results:**

341 patients (82 LSD, 116 MSD and 143 HSD) were included in the study (mean follow up of 1.5 years) with a mean eGFR decline of 2.7 ml/min/1.73 m^2^/year. 105 patients (31 %) required renal replacement therapy and 10 (3 %) died. There was no association between urinary sodium excretion and change in the eGFR or need for renal replacement therapy and mortality in crude or adjusted models (unadjusted HR 1.002; 95%CI 1.000–1.004, adjusted HR 1.001; 95%CI 0.998–1.004).

**Conclusion:**

In patients with advanced CKD (eGFR < 30 ml/min/1.73 m^2^), sodium intake does not appear to impact the progression of CKD to end-stage renal disease; however, more definitive studies are needed.

**Electronic supplementary material:**

The online version of this article (doi:10.1186/s12882-016-0338-z) contains supplementary material, which is available to authorized users.

## Background

Patients with chronic kidney disease (CKD) have a significantly higher mortality compared to the general population and this increases as the estimated glomerular filtration rate (eGFR) declines [1]. Thus, preservation of kidney function and avoidance of end-stage renal disease (ESRD) is a key therapeutic target. Among the recommendations for preservation of kidney function is the control of dietary sodium as a modifiable risk factor.

Recent international guidelines have incorporated sodium restriction into their recommendations. Kidney Disease Improving Global Outcomes (KDIGO) suggests a reduction to <2 g/day of sodium which corresponds to 5 g/day of salt for adult patients with CKD [2]. These recommendations are based on low level evidence from studies with marked heterogeneity [3], although the results overall suggest that modest sodium restriction should be beneficial for patients with CKD. However, there is a paucity of studies specifically addressing the effect of sodium intake in patients with advanced CKD (defined as eGFR < 30 ml/min/1.73 m^2^). Studies that included individuals with advanced CKD have been limited by surrogate short-term outcomes or inclusion of non-diabetic patients [4–6]. As such, in patients with advanced CKD, it remains unknown whether alterations in sodium intake impact clinically relevant outcomes, such as mortality, CKD progression and the need for dialysis.

The aim of our study was to determine if urinary sodium excretion is associated with mortality and need for renal replacement therapy in patients with advanced CKD. We hypothesized that higher levels of urinary sodium excretion (used as a surrogate for sodium intake) would be associated with adverse clinical outcomes.

## Methods

### Patient population and measurements

This is a retrospective cohort study using prospectively collected data on adult patients (>18 years) followed in the progressive renal insufficiency clinic at The Ottawa Hospital, a 1,150 bed academic tertiary care center serving a population of approximately 1.2 million, located in Ontario, Canada. The progressive renal insufficiency clinic is a specialty multi-disciplinary care clinic in the CKD program for patients approaching ESRD. Patients with progressive kidney disease are referred to this clinic at the discretion of primary nephrologist in anticipation of needing renal replacement therapy. Attempts are made to collect 24 h urine for sodium excretion at least twice per year. Standardised instructions are given to each patient for collection of 24 h urine.

The study included patients with eGFR < 30 ml/min/1.73 m^2^ calculated by the 4 variable MDRD formula [7], with at least two visits to the clinic and at least one 24-h urine sodium excretion (USE) measurement between January 2010 and December 2012. Patients who required any kind of renal replacement therapy prior to the second visit were excluded. The date of first visit was deemed the date of study entry.

### Data collection

Data on all patients were obtained through the clinic’s comprehensive database since its inception in January 1st, 2010. The accuracy of the data in the database is verified every six months by auditing 5 % of entries and accuracy has been > 95 %. Data collected included age, gender, blood pressure (BP), body-mass index (BMI), eGFR, urine excretion of sodium, protein and creatinine, cause of CKD, comorbidities, medications, date of death and date of dialysis start or renal transplantation. Blood pressure was measured in sitting position after at least five minutes of rest. Mean arterial blood pressure (MAP) was calculated using the formula: MAP = 2/3 diastolic BP + 1/3 systolic BP. Urine sodium, urine creatinine and urine protein were all measured on the Siemens Vista 1500 analyzer using manufacturers’ reagents. If 24- h urine protein was not available, spot urine protein to creatinine ratios were converted to 24 h urine protein excretion [8].

Blood pressure, eGFR and proteinuria were measured at each clinic visit. 24-h USE was performed periodically to monitor compliance and assist in counseling patients regarding sodium restriction.

Sodium intake for each patient was estimated from the average 24-h USE measured during the follow-up period. In order to account for inappropriate collection of 24-h urine, 24-h urine sodium excretion was corrected for expected creatinine excretion. Expected creatinine excretion was calculated by the following formulas: If male then creatinine excretion (mmol/day) = (28.2−(0.172*age))* 0.00884*weight (Kg); if female then creatinine excretion (mmol/day) = (21.9−(0.115*age))* 0.00884*weight (Kg) [9]. *A priori*, we categorized the patients according to their mean 24-h USE into three groups: low sodium diet (LSD) -USE < 100 mEq/day, medium sodium diet (MSD) - USE 100–150 mEq/day and high sodium diet (HSD) - USE > 150 mEq/day. We selected these cut-off points first, to determine whether patients with advanced CKD have higher mortality or faster progression of CKD if their sodium intake is higher than currently recommended (>100 mEq/day), and second, to investigate whether daily sodium intake between 100 and 150 mEq (which is simpler to achieve than <100 mEq/day for most patients) is associated with different outcomes compared to LSD and HSD.

### Outcomes

The primary outcome was a composite of progression to ESRD requiring any type of renal replacement therapy (dialysis or renal transplant) and mortality.

The secondary outcome was the decline in eGFR in ml/min/1.73 m^2^ per year.

### Statistical analyses

Continuous variables are reported as mean (± SD) and categorical variables are reported as numbers and percentages. Analysis of variance with Tukey correction was used to compare continuous variables. If Levene's test for equality of error variances was positive, the data was log transformed. Chi squared test was used to compare categorical variables. Time-to-event analyses were conducted using the Kaplan-Meier (KM) method and Cox proportional hazards. The log rank test was used with the KM method to assess for statistical significance. To better quantify individual sodium exposure and account for within person data variability, sodium intake was modeled continuously using time-dependent Cox models using the mean 24-h urine sodium excretion. Potential confounders included in the Cox models were sex, age, baseline eGFR, mean arterial blood pressure (MAP), body mass index (BMI), diabetes, log transformed 24-h proteinuria, and renin-angiotensin system (RAS) blockers (angiotensin converting enzyme inhibitors and angiotensin receptor blockers) use. MAP and log transformed 24-h proteinuria were treated as time dependent variables, and changes in their values over time were incorporated into the adjusted Cox analyses. Sensitivity analysis was performed for sodium uncorrected for expected creatinine excretion as well as after censoring for death.

All statistical analyses were performed using SAS software (Version 9.3) and Medcalc (Version 13.2). *P* < 0.05 was considered statistically significant.

## Results

### Baseline characteristics

The baseline characteristics of the study cohort are presented in Table 1. Three hundred forty-one patients were included with a mean age of 64.4 years. Most patients were male (62.5 %) and had an elevated BMI (30 kg/m^2^). At baseline, MAP was 111 mmHg and the mean eGFR was 17.1 ml/min/1.73 m^2^. The most common cause of CKD was diabetic nephropathy. The majority of patients were hypertensive and diabetic (94 % and 58 % respectively). 53 % were on RAS blockade.

**Table 1:** Baseline characteristics

	All *N* = 341	LSD *N* = 82	MSD *N* = 116	HSD *N* = 143	*P* value
Age years, mean (SD)	64.4 (14.9)	68.4 (15.1) ^β^	66.9 (15.4)	60.1 (13.2)^μ^	<0.05
Female, n (%)	128 (37.5)	35 (42.7)	46 (39.7)	47 (32.9)	0.29
MAP mm Hg, mean (SD)	111 (14.2)	111 (14.1)	114 (15.1)	112 (13.5)	0.36
BMI kg/m^2^, mean (SD)	29.9 (6.6)	25.3 (4.1) ^α, β^	28.6 (4.8) ^μ,^	33.7 (7.0)	<0.05
eGFR ml/min/1.73 m2 mean, (SD)	17.1 (5.5)	16.4 (4.1)	18.0 (6.7)	16.7 (5.2)	0.30
Urine protein, g/day median, (IQR)	1.5 (2.7)	1.0 (2.1) ^α, β^	1.3 (1.9)	1.9 (3.4)	<0.05
*Cause of CKD, n (%)*					
Diabetic nephropathy	162 (47.5)	27 (32.9)	50 (43.1)	85 (59.4)	0.06
Ischemic nephropathy	61 (17.9)	21 (25,6)	21 (18.1)	19 (13.3)	0.16
Glomerulonephritis	43 (12.7)	14 (17.1)	16 (13.8)	13 (9.1)	0.29
PCKD	27 (8)	8 (9.6)	10 (8.6)	9 (6.3)	0.67
Other	48 (14.1)	12 (14.6)	19 (16.4)	17 (11.9)	0.66
*Comorbidities, n (%)*					
Diabetes	198 (58.2)	32 (39)	61 (52.6)	105 (73.4)	0.02
CAD	105 (30.8)	20 (24.4)	35 (30.2)	50 (35.0)	0.48
CHF	79 (23.2)	13 (15.9)	22 (19)	44 (30.8)	0.08
Hypertension	319 (93.6)	76 (92.7)	109 (94)	134 (93.7)	0.99
CVA	36 (10.6)	7 (8.5)	12 (10.3)	17 (11.9)	0.78
Dyslilidemia	235 (68.9)	50 (61)	81 (69.8)	104 (72.7)	0.72
*Medications, n (%)*					
RAS blocker	181 (53.1)	40 (48.8)	64 (55.2)	77 (53.9)	0.88
Diuretic	219 (64.2)	48 (58.5)	76 (65.5)	95 (66.4)	0.84
Calcium channel blockers	237 (69.5)	53 (64.6)	87 (75)	97 (67.8)	0.78
Beta blockers	178 (52.2)	38 (46.3)	62 (53.4)	78 (54.5)	0.78

*SD* standard deviation, *IQR* interquartile range, *MAP* mean arterial blood pressure, *BMI* body mass index, *eGFR* estimated glomerular filtration rate, *PCKD* polycystic kidney disease, *CAD* coronary artery disease, *CHF* congestive heart failure, *CVA* cerebrovascular accident, RAS renin-angiotensin system

^α^P < 0.05 between LSD and MSD

^β^P < 0.05 between LSD and HSD

^μ^P < 0.05 between MSD and HSD

Based on the mean USE during the observational period, 82, 116, and 143 patients were categorized into low sodium diet (LSD; USE < 100 mEq/day), medium sodium diet (MSD; USE 100–150 mEq/day), and high sodium diet (HSD; USE > 150 mEq/day) groups, respectively. At baseline, the three groups did not differ in gender, MAP, eGFR, or use of diuretics and RAS blockers. Patients from the HSD group were younger (mean age 60 years) with a higher BMI (33.7 kg/m^2^) and were more proteinuric. The variability in sodium excretion in patients with more than one 24-h urine collection was assessed by intra-individual coefficient of variation (CV) defined as SD/Mean. The CV for the whole group was 55 %. We also assessed variability in sodium excretion by dividing patients into two groups by median of baseline eGFR and found that CV for the higher eGFR group was 56 % and for the lower eGFR group it was 53 % indicating similar variability in sodium excretion in the two groups.

### Sodium diet groups and outcomes

The mean follow-up time was 1.5 years. The average number of 24-h USE measurements per patient was 3.6 (±2.0). 54 patients had only one sodium measurement. The three groups differed in mean USE as expected (*P* < 0.01), however, there was no significant difference in eGFR decline, RRT requirement or death (Table 2). A sensitivity analysis comparing LSD to all other patients also did not reveal a statistically significant difference; (Additional file 1 and 2). A sensitivity analysis using CKD-EPI eGFR also did not change the results (Additional file 3).

**Table 2:** Cohort characteristics at the end of follow-up

	All	LSD	MSD	HSD	*P* value
Duration of F/U years, mean (SD)	1.5 (0.91)	1.5 (0.88)	1.6 (0.91)	1.47 (0.93)	0.54
^α^24-h USE mEq/day, mean (SD)	156 (86)	79 (23)	124 (31)	222 (87)	<0.01
Change in eGFR/year, ml/min/1.73 m^2^; mean (SD)	2.7 (5.0)	2.51 (5.2)	3.36 (5.5)	3.33 (6.4)	0.55
ESRD n (%)	105 (30.8)	22 (26.8)	35 (30.2)	48 (33.6)	0.74
Deaths n (%)	10 (2.9)	3 (3.7)	3 (2.6)	4 (2.8)	0.91

*SD* standard deviation, *F/U* follow-up, *USE* urinary sodium excretion, *eGFR* estimated glomerular filtration rate, *ESRD* end stage renal disease

^**α**^Corrected for creatinine excretion

### Time-to-event (ESRD + death) analyses

**Fig. 1:**
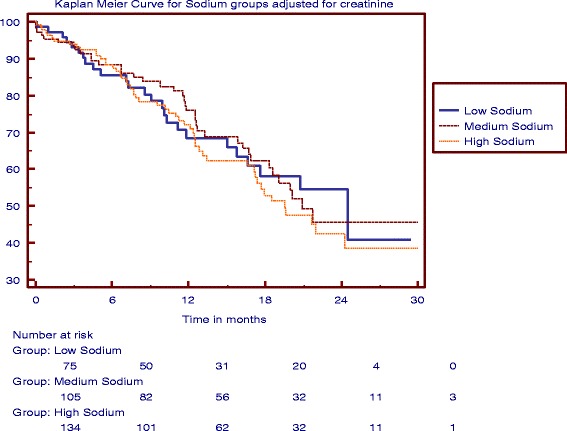
Kaplan Meier Curve for Sodium Groups. The analysis did not show any difference in time to composite outcome (renal replacement therapy + Death) among the three groups (*P* = 0.73)

Of the 115 patients (34 %) who reached the primary outcome (105 reached ESRD and 10 died), 25 (31 %) were from the LSD group, 38 (33 %) from the MSD group, and 52 (36 %) from the HSD group (Table 2). The median time to RRT and death among the three groups was 21 months (95 % CI, 18.3–25) and it did not differ significantly (Log rank test; *P* = 0.73; Fig. 1).

In the unadjusted Cox analysis that modeled USE as a continuous variable, increase in USE was not significantly associated with an excess risk of progression to ESRD and mortality (HR 1.002; 95 % CI 1.000–1.004; (Table 3). After adjusting for age, sex, baseline eGFR, MAP, proteinuria, diabetes, BMI, and RAS blockers, the hazard ratio for need for RRT and mortality did not change (Table 3). A sensitivity analyses conducted after censoring death did not change the results (Additional file 4). A second sensitivity analysis conducted with sodium excretion uncorrected for expected creatinine excretion also did not change the results (Additional file 5). Since MAP is likely to be on the causal pathway for the association between sodium and renal outcomes, we did another sensitivity analysis by excluding MAP from the Cox model for primary outcome and results did not change significantly (Additional file 6).

**Table 3:** Cox model for primary outcome (renal replacement therapy + death)

	Hazard ratio	95 % CI	*P* value
*Unadjusted*	1.002	1.000 – 1.004	0.07
*Multivariable adjusted*			
Urinary sodium excretion	1.001	0.998 – 1.004	0.51
Age	1.005	0.992 – 1.018	0.47
Female	0.507	0.339 – 0.758	<0.01
Baseline eGFR	0.832	0.782 – 0.885	<0.01
MAP over time	1.005	0.992 – 1.018	0.45
Log proteinuria over time	1.646	1.325 – 2.045	<0.01
Diabetes	0.86	0.58 – 1.268	0.44
BMI	0.959	0.921 – 0.998	0.04
RAS blockers	0.839	0.569 – 1.236	0.37

*MAP* mean arterial blood pressure, *RAS* renin angiotensin system, *BMI* body mass index

## Discussion

In this retrospective cohort study of 341 patients with advanced CKD, we did not find an association between urinary sodium excretion (USE) and combined outcome of mortality and need for renal replacement therapy (RRT) or eGFR decline. Patients with high USE were more likely to be younger, had higher BMI, and were more proteinuric. There was no association between need for RRT and the mean USE as a categorical or continuous variable. Furthermore, there was no association between USE and the eGFR decline. Our study suggests USE (a surrogate for dietary sodium) may not play a significant role in the progression of CKD to end-stage renal disease in patients with severe kidney disease. To our knowledge, this is the first report on the association of USE on the need for renal replacement therapy and eGFR declines in a diverse CKD population with eGFR < 30 ml/min/1.73 m^2^.

There are no randomized control trials (RCTs) examining sodium intake and long-term renal outcomes, such as progression of CKD or ESRD and thus most of the evidence comes from observational studies. There are, however, short-term RCTs showing a beneficial effect of dietary sodium restriction on proteinuria and blood pressure [6, 10]. This can suggest that lowering sodium intake may slow down the eGFR decline and thus postpone dialysis initiation. The Modified Diet in Renal Disease (MDRD) study that included 840 non-diabetic patients with CKD (mean eGFR 32.5 ml/min/1.73 m^2^) did not show any association between urine sodium and kidney failure (which was defined as initiation of dialysis or transplantation) or the composite outcome of renal failure and all-cause mortality [5]. Neither a recent post hoc analysis of the ONTARGET and TRANSCEND trials revealed an association between sodium intake and clinically important renal outcomes in patients with cardio-vascular risk factors [11]. A limitation of these two trials was that the sodium intake was estimated from a spot morning urine sample using the Kawasaki formula, however, the large sample size (28, 880 patients), international representation of the cohort and completeness of data retrieval add considerable robustness to the conclusions. A secondary analysis of the REIN trials [12] involving 500 non-diabetic patients with CKD (baseline creatinine clearance 42.5 ml/min) demonstrated that each 100 mEq/g increase in urinary sodium/creatinine excretion was associated with a 1.61-fold (95 % CI, 1.15–1.24) higher risk for ESRD. However, after adjusting for baseline proteinuria this effect was significantly attenuated to 1.38-fold (95 % CI; 0.95–2.00). A recent study by McQuarrie et al [13] showed a similar finding – they demonstrated an association between USE and the risk of requiring RRT in CKD patients, but this was not independent of albuminuria or baseline eGFR.

More than half of the patients involved in our report had diabetes. The Finn Diane study that followed 2,807 type I diabetics with mean baseline eGFR > 80 ml/min/1.73 m^2^ over 10 years, showed an inverse association of urine sodium and ESRD – macroalbuminuric patients with the lowest sodium excretion had the highest cumulative incidence of ESRD. [14] The post-hoc analysis of RENAAL and IDNT yielded different results [15]. The authors reported a significantly lower risk for doubling of serum creatinine or ESRD in the lowest tertile of sodium intake if the patients were on angiotensin receptor blocker treatment, compared to the control group treated with non-RAS antihypertensive agents. Of note, sodium intake was not randomized and there were significant differences in the study population characteristics such as lower eGFR and higher urinary albumin-to-creatinine ratio in the highest tertile of sodium intake (for which the analysis was not adjusted).

In our CKD study population, 58 % of patients (LSD and MSD group) had sodium intake within or close to the recommended target with HSD group represented a common salt intake of the general population in western and particularly in Asian countries [16]. In spite of clear separation of the groups, we did not find an effect of lower sodium excretion resulting in less mortality and need for ESRD. It is also possible that sodium intake does not play a significant role in CKD progression once renal disease is very advanced. Another possible factor is salt sensitivity that as a mechanism may be different in CKD populations to the general population. Finally, marked dietary sodium restriction may have adverse consequences, including activation of the sympathetic and RAAS systems, reduction in insulin sensitivity, and increased LDL cholesterol [17, 18]. Indeed, the relationship between sodium intake and mortality in the general population is J-curved [19], and it is possible that a similar relationship exists for renal disease progression; although the nadir may vary in different patient populations.

Our study has two strengths: first, the data were obtained from a CKD patient population who received treatment based on current guidelines. Second, although it is an observational study, the data was prospectively collected and data entry was verified periodically with > 95 % of data being accurate.

The major study limitation was short follow-up period. Although there is a trend to more ESRD and faster GFR decline with higher sodium intake, it is possible that follow-up was not long enough to demonstrate statistically significant impact on ESRD and GFR decline. The second limitation is the observational nature of the study, thus causal relationships cannot be discerned, and residual confounding could impact the results. Although we calculated the average of urinary sodium excretion for each patient, the number of measurements per patient was low (mean 3.4 USE measurements/patient), and therefore we cannot account for changes in sodium intake that occurred before or after urinary sodium testing. Moreover, there are day-to-day variability in sodium intake and excretion [20], which may also affect the accuracy of sodium intake estimation. The timing of initiating diuretics and increasing their dose may also impact sodium excretion. At our institution, diuretics are usually initiated (or dose changed) at the visit and urine collection is done on a subsequent visit thus minimizing the impact of change in diuretic dose on sodium excretion. In addition we could not address the issue of mortality with high sodium intake because of low number of deaths in our population. Finally, In our study RAS blockers were used more often in patients who were in the MSD and HSD group. Thus in our study, overweight and diabetic patients would have benefited less from RAS blockers.

## Conclusion

In patients with eGFR < 30 ml/min/1.73 m^2^, sodium intake did not affect progression of CKD to dialysis. Given the limitations of our study outlined above, prospective studies are desirable to address the issue of appropriate counseling for sodium intake in patients with advanced CKD.

## Additional files

Additional file 1: Low sodium diet (LSD) versus combination of medium (MSD) + High sodium diet (HSD). (DOC 29 kb) Additional file 2: The analysis did not show any difference in time to composite outcome (renal replacement therapy + Death) between Low Sodium Diet (LSD) and Medium (MSD) + High Sodium Diet (HSD) (*P*=0.81). (DOC 28 kb) Additional file 3: Change in CKD- EPI eGFR per year. (DOC 27 kb) Additional file 4: Cox model for renal replacement therapy only. (DOC 32 kb) Additional file 5: Cox model for primary outcome (renal replacement therapy + death) – urinary sodium excretion not corrected for expected creatinine excretion. (DOC 31 kb) Additional file 6: Cox model for primary outcome (renal replacement therapy + death) excluding Mean arterial pressure. (DOC 31 kb)

## Abbreviations

BMI: Body mass index
BP: Blood pressure
CKD: Chronic kidney disease
eGFR: estimated glomerular filtration rate
ESRD: End-stage renal disease
HSD: High sodium diet
LSD: Low sodium diet
MAP: Mean arterial pressure
MDRD: Modified Diet in Renal Disease
MSD: Moderate sodium diet
RAS: Renin-angiotensin system
RRT: Renal replacement therapy
