# Supplementary material for: The association of urinary sodium excretion and the need for renal replacement therapy in advanced chronic kidney disease: a cohort study
Source: BMC Nephrol. 2016 Sep 5;17(1):123. doi: 10.1186/s12882-016-0338-z (PMC5011929; doi:10.1186/s12882-016-0338-z)
Supplement: Additional file 1: — Low sodium diet (LSD) versus combination of medium (MSD) + High sodium diet (HSD). (DOC 29 kb) [file 12882_2016_338_MOESM1_ESM.doc]

Additional File 1:

Low sodium diet (LSD) versus combination of medium (MSD) + High sodium diet (HSD)

|  | All | LSD | MSD & HSD | P value |
| --- | --- | --- | --- | --- |
| Change in eGFR/year, ml/min/1.73m^2^; mean (SD) | 2.7 (5.0) | 2.51 (5.2) | 3.35 (5.1) | 0.26 |
| ESRD n (%) | 105 (30.8) | 22 (26.8) | 83 (32.1) | 0.37 |
| Deaths n (%) | 10 (2.9) | 3 (3.7) | 7 (2.8) | 0.68 |

eGFR- estimated glomerular filtration rate, ESRD- end stage renal disease
