# Supplementary material for: The association of urinary sodium excretion and the need for renal replacement therapy in advanced chronic kidney disease: a cohort study
Source: BMC Nephrol. 2016 Sep 5;17(1):123. doi: 10.1186/s12882-016-0338-z (PMC5011929; doi:10.1186/s12882-016-0338-z)
Supplement: Additional file 2: — The analysis did not show any difference in time to composite outcome (renal replacement therapy + Death) between Low Sodium Diet (LSD) and Medium (MSD) + High Sodium Diet (HSD) (P=0.81). (DOC 28 kb) [file 12882_2016_338_MOESM2_ESM.doc]

Additional file 2: The analysis did not show any difference in time to composite outcome ( renal replacement therapy + Death) between Low Sodium Diet (LSD) and Medium (MSD) + High Sodium Diet (HSD) (P=0.81).
