# Supplementary material for: The association of urinary sodium excretion and the need for renal replacement therapy in advanced chronic kidney disease: a cohort study
Source: BMC Nephrol. 2016 Sep 5;17(1):123. doi: 10.1186/s12882-016-0338-z (PMC5011929; doi:10.1186/s12882-016-0338-z)
Supplement: Additional file 3: — Change in CKD- EPI eGFR per year. (DOC 27 kb) [file 12882_2016_338_MOESM3_ESM.doc]

Additional File 3:

Change in EPI-eGFR per year

|  | All | LSD | MSD | HSD | P value |
| --- | --- | --- | --- | --- | --- |
| Change in EPI-eGFR/year, ml/min/1.73m^2^; mean (SD) | 3.1 (6.1) | 2.47 (5.2) | 3.21 (5.6) | 3.38 (6.8) | 0.55 |

eGFR – estimated glomerular filtration rate
