# Supplementary material for: The association of urinary sodium excretion and the need for renal replacement therapy in advanced chronic kidney disease: a cohort study
Source: BMC Nephrol. 2016 Sep 5;17(1):123. doi: 10.1186/s12882-016-0338-z (PMC5011929; doi:10.1186/s12882-016-0338-z)
Supplement: Additional file 4: — Cox model for renal replacement therapy only. (DOC 32 kb) [file 12882_2016_338_MOESM4_ESM.doc]

Additional File 4:

Cox model for renal replacement therapy only

|  | Hazard ratio | 95% CI |
| --- | --- | --- |
| Urinary Sodium Excretion - Unadjusted | 1.001 | 0.997-1.004 |
| *Multivariable adjusted* |  |  |
| Urinary Sodium Excretion | 0.998 | 0.994-1.002 |
| Age | 0.996 | 0.985-1.008 |
| Female | **0.543** | **0.356-0.830** |
| Baseline eGFR | **0.81** | **0.755-0.869** |
| MAP over time | 1.008 | 0.995-1.022 |
| Log proteinuria over time | **1.813** | **1.440-2.282** |
| Diabetes | 0.798 | 0.530-1.204 |
| BMI | 0.974 | 0.935-1.013 |
| RAS blockers | 0.870 | 0.576-1.314 |

eGFR – estimated glomerular filtration rate, MAP – mean arterial blood pressure, BMI – body mass index, RAS – renin- angiotensin system
