# Supplementary material for: The association of urinary sodium excretion and the need for renal replacement therapy in advanced chronic kidney disease: a cohort study
Source: BMC Nephrol. 2016 Sep 5;17(1):123. doi: 10.1186/s12882-016-0338-z (PMC5011929; doi:10.1186/s12882-016-0338-z)
Supplement: Additional file 5: — Cox model for primary outcome (renal replacement therapy + death) – urinary sodium excretion not corrected for expected creatinine excretion. (DOC 31 kb) [file 12882_2016_338_MOESM5_ESM.doc]

Additional File 5:

Cox model for primary outcome (renal replacement therapy + death) – urinary sodium excretion not corrected for expected creatinine excretion

|  | Hazard Ratio | 95% CI |
| --- | --- | --- |
| Urinary Sodium Excretion - Unadjusted | 1.000 | 0.997-1.004 |
| *Multivariable adjusted* |  |  |
| Urinary Sodium Excretion | 0.998 | 0.994-1.002 |
| Age | 1.001 | 0.989-1.013 |
| Female | **0.494** | **0.330-0.738** |
| Baseline eGFR | **0.831** | **0.781-0.884** |
| MAP over time | 1.003 | 0.991-1.016 |
| Log proteinuria over time | **1.687** | **1.362-2.090** |
| Diabetes | 0.891 | 0.603-1.317 |
| BMI | 0.970 | 0.935-1.007 |
| RAS blockers | 0.84 | 0.572-1.235 |

eGFR – estimated glomerular filtration rate, MAP – mean arterial blood pressure, BMI – body mass index, RAS – renin- angiotensin system
