# Supplementary material for: The association of urinary sodium excretion and the need for renal replacement therapy in advanced chronic kidney disease: a cohort study
Source: BMC Nephrol. 2016 Sep 5;17(1):123. doi: 10.1186/s12882-016-0338-z (PMC5011929; doi:10.1186/s12882-016-0338-z)
Supplement: Additional file 6: — Cox model for primary outcome (renal replacement therapy + death) excluding Mean arterial pressure. (DOC 31 kb) [file 12882_2016_338_MOESM6_ESM.doc]

Additional File 6:

Cox model for primary outcome (renal replacement therapy + death) excluding Mean arterial pressure

|  | **Hazard ratio** | **95% CI** |
| --- | --- | --- |
| *Urine Sodium Excretion-Unadjusted* | 1.002 | 1.000 – 1.004 |
| *Multivariable adjusted* |  |  |
| Urinary sodium excretion | 1.001 | 0.998 – 1.004 |
| Age | 1.004 | 0.992 – 1.017 |
| Female | **0.498** | **0.333 – 0.743** |
| Baseline eGFR | **0.825** | **0.774 – 0.878** |
| Log proteinuria over time | **1.698** | **1.360 – 2.119** |
| Diabetes | 0.82 | 0.551 – 1.220 |
| BMI | 0.957 | **0.920 – 0.997** |
| RAS blockers | 0.85 | 0.577 – 1.253 |

eGFR – estimated glomerular filtration rate, BMI – body mass index, RAS – renin- angiotensin system
